# Better transplant outcome with pre-transplant marrow response after hypomethylating treatment in higher-risk MDS with excess blasts

**DOI:** 10.18632/oncotarget.12511

**Published:** 2016-10-06

**Authors:** Seung-Ah Yahng, Myungshin Kim, Tae-Min Kim, Young-Woo Jeon, Jae-Ho Yoon, Seung-Hwan Shin, Sung-Eun Lee, Ki-Seong Eom, Seok Lee, Chang-Ki Min, Hee-Je Kim, Dong-Wook Kim, Jong-Wook Lee, Woo-Sung Min, Yoo-Jin Kim

**Affiliations:** ^1^ Incheon St. Marys Hospital, College of Medicine, The Catholic University of Korea, Incheon, Korea; ^2^ Department of Laboratory Medicine, Seoul St. Marys Hospital, College of Medicine, The Catholic University of Korea, Seoul, Korea; ^3^ Leukemia Research Institute, College of Medicine, The Catholic University of Korea, Seoul, Korea; ^4^ Department of Medical Informatics, College of Medicine, The Catholic University of Korea, Seoul, Korea; ^5^ Catholic Blood and Marrow Transplantation Center, Seoul St. Marys Hospital, College of Medicine, The Catholic University of Korea, Seoul, Korea; ^6^ Yeoido St. Marys Hospital, College of Medicine, The Catholic University of Korea, Seoul, Korea

**Keywords:** higher-risk myelodysplastic syndrome, marrow response, hypomethylating treatment, allogeneic hematopoietic stem cell transplantation

## Abstract

Hypomethylating treatment (HMT) has been suggested as a feasible bridge to hematopoietic stem cell transplantation (HSCT), but controversies exist around influences of HMT response on transplant outcomes. To assess the safety and influences of pre-transplant HMT focusing on debulking effects and transplant outcomes, we retrospectively analyzed consecutive HSCT-eligible patients who received HMT for higher-risk MDS with excess blasts. Of all 98 patients, 11 patients failed to proceed to HSCT and HMT-related mortality occurred in 8 patients. When excluding 9 patients who refused HSCT, 87% of scheduled HSCT (77 of 89) was performed after a median of 3 cycles (range, 1-8) of HMT. The 4-year overall survival after HMT (*n* = 98) and HSCT (*n* = 77) was 44.0% and 53.6%, respectively. Transplant outcomes were significantly different by the final response at HSCT; marrow response group (complete remission, marrow complete remission with or without hematologic improvement) showed significantly better 4-year disease-free survival compared to no marrow response group (*n* = 36, 87.3% *vs*. *n* = 41, 10.7%, *P* < 0.001). This difference between the groups was also evident in overall survival (90.9% *vs*. 8.6%, *P* < 0.001) and cumulative incidences of relapse (6.5% *vs*. 45.4%, *P* < 0.001) and treatment-related mortality (6.2% *vs*. 43.9%, *P* < 0.001). These observations indicate that pre-transplant HMT is a feasible bridging treatment in patients with excess blasts regarding high success rate of proceeding to transplantation and good survival rate. Marrow response at HSCT regardless of concomitant hematological improvement is an independent predictor of better survival, suggesting that immediate HSCT rather than continuing HMT should be performed once marrow response is achieved.

## INTRODUCTION

Allogeneic hematopoietic stem-cell transplantation (HSCT) remains the only curative strategy to treat patients with myelodysplastic syndromes (MDS), and the role of bridging therapy using intensive chemotherapy or hypomethylating agents followed by HSCT in higher-risk MDS has been suggested [1]. Advances in the uses of alternative donors and reduced intensity conditioning regimens have extended the use of allogeneic HSCT to a wider number of patients, while high-resolution HLA typing and better supportive care facilitated minimizing treatment-related toxicities in older and less fit patients [2, 3].

However, the efficacy of HSCT in higher-risk MDS remains unsatisfactory, compared to that in lower-risk MDS, due to the high rates of relapse (30-40%) and treatment-related mortality (TRM; 30-50%), leading to worse long-term disease-free survival (DFS; < 30% for higher-risk MDS) [4–6]. To achieve successful pre-transplant disease control, as well as reduce post-transplant relapse, pre-transplant intensive chemotherapy has been tried for patients with higher-risk MDS with excess blasts who are particularly at risk of brisk disease progression (DP) and transformation to acute myeloid leukemia (AML) [6, 7].

Feasibility of pre-transplant hypomethylating treatment (HMT) has been suggested in several previous reports [8–11]. HMT is currently a standard therapy for aged or debilitated patients with higher-risk MDS by its favorable toxicity profile [1, 12] and substantial response rate even in patients with poor cytogenetics [13–14]. In our previous report, the positive effect of HMT response on post-transplant survival was mostly due to marrow response in patients with higher-risk MDS [11], suggesting that the influences of HMT response might be associated with debulking effects of HMT. Currently, there has been little research on the use of HMT for “debulking strategy” focusing on higher-risk MDS with excess marrow blasts at HMT; thus, the issue as to whether HMT could be an alternative to induction chemotherapy in the setting remains to be clarified.

Given the above, this retrospective study was designed to assess the role of pre-HSCT HMT by analyzing treatment toxicities, rates of proceeding to HSCT, and influences of response type (especially marrow response) on transplant outcomes. To minimize selection bias, we included all consecutive HSCT-eligible patients who received HMT for higher-risk MDS with excess blasts.

## RESULTS

### Baseline characteristics at pre-transplant HMT

Ninety-eight patients (61 men and 37 women) with higher-risk MDS [15] and excess marrow blasts eligible for HSCT had received azacitidine (*n* = 66) or decitabine (*n* = 32). HMT was initiated from a median of 20 days (range, 1-180 days) after the diagnosis of higher-risk MDS. The median age of patients was 53 years (range, 18-65 years). The cytogenetic risk was good in 44 cases (45%), intermediate in 29 cases (30%) and poor in 25 cases (25%), and the median percentage of bone marrow blast at HMT was 13 (range, 6-19).

### Response and disease course after pre-transplant HMT

**Figure 1:**
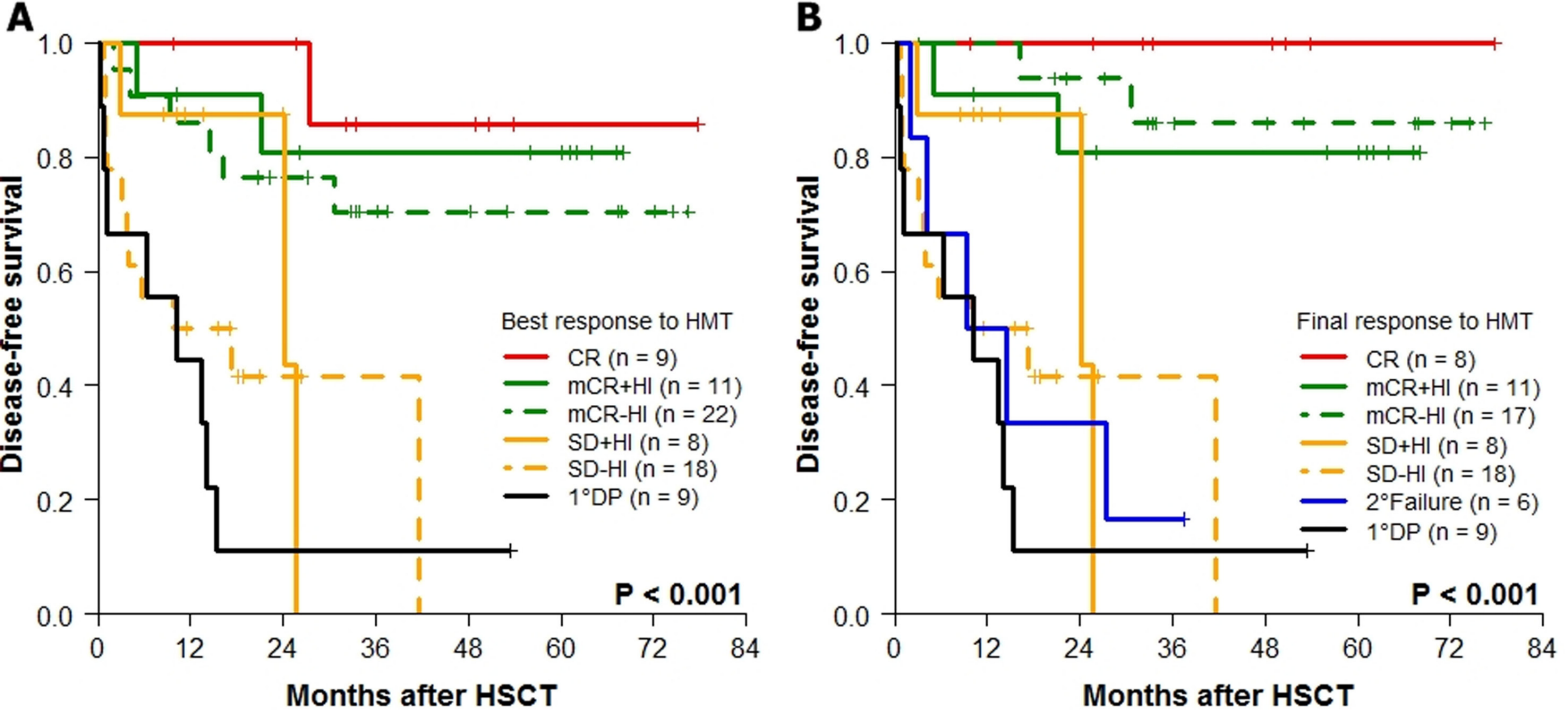
Response and survival from HMT initiation in higher-risk MDS patients with excess blasts **A**. Summary of clinical responses during HMT according to the four groups observed: one patient in preparation for HSCT, 77 patients undergoing HSCT, 11 patients who became HSCT ineligible (HSCT failure), and 9 patients who refused to receive HSCT. Kaplan-Meier survival from HMT of **B**. all 98 patients, **C**. HSCT group, **D**. HSCT failure group, and **E**. HSCT refusal group.

Of all 98 study patients, 59 patients (60%) showed a treatment response after a median of 2 cycles (range, 1-9 cycles) of HMT, and their best responses were complete remission (CR, *n* = 12), marrow CR with hematologic improvement (mCR+HI, *n* = 12), mCR without HI (mCR-HI, *n* = 26), and stable disease with HI (SD+HI, *n* = 9; Figure 1A). Eleven (11%) patients became HSCT-ineligible (HSCT failure), and HMT-related mortality occurred in 8 of 98 patients (8.2%). Nine (9%) patients refused HSCT, while 1 patient was in its preparation at our data cut-off point. When excluding 9 patients who refused HSCT, 87% of scheduled HSCT (77 of 89 patients) was performed after a median of 3 cycles (range, 1-8) of HMT. Responses at the time of HSCT revealed that 44 patients (57%) continuously maintained their best responses to HMT (continued response), 27 patients (35%) never responded to HMT (primary failure; SD-HI, *n* = 17; primary DP, *n* = 10), and 6 patients (8%) lost their best responses (secondary failure; relapse from CR, *n* = 1; relapse from mCR-HI, *n* = 5). AML-like induction chemotherapy was administered in 9 of the 11 patients with AML transformation (primary DP to AML, *n* = 10; relapse in AML from mCR-HI, *n* = 1). Further details are illustrated in Figure 1A.

The median overall survival (OS) for all patients from the initiation of HMT was 39.3 months (95% confidence interval [CI]: 23.9-54.6 months; Figure 1B), whereas the OS for the patients who received a transplant did not reach the median (Figure 1C). The median OS for patients in HSCT failure and those in HSCT refusal were 4.3 months (95% CI: 0.6-8.1 months; Figure 1D) and 15.8 months (95% CI: 12.4-19.1 months; Figure 1E), respectively.

### Outcomes of transplantation following pre-transplant HMT

Seventy-five of the 77 transplant patients (97.4%) achieved primary engraftment, with median times to neutrophil and platelet engraftment of 12 days (range: 10-23 days) and 16 days (range: 9-51 days), respectively, while 2 patients died before neutrophil recovery could occur 22 and 24 days after graft infusion. The cumulative incidence of 100-day grade II-IV acute graft-versus-host disease (GVHD) was 29.9% ± 5.3%, and that of 2-year chronic GVHD among the evaluable patients (*n* = 73) was 48.1% ± 5.9%. After a median follow-up of 41.2 months (95% CI: 25.3-57.6 months) among the survivors, 47 patients are currently alive in remission (*n* = 46) or in relapse (*n* = 1), and 30 patients have died due to relapse (*n* = 15) or TRM (*n* = 15). The causes of TRM were extensive chronic GVHD (*n* = 4), grade IV acute GVHD (n = 4), and infection (*n* = 7). The 4-year probabilities of DFS and OS after HSCT were 53.6% ± 6.5% and 53.8% ± 6.6%, respectively, and the 4-year cumulative incidence of relapse (CIR) and TRM (CITRM) were 23.9% ± 5.4% and 22.5% ± 5.4%, respectively.

### Effect of HMT response at HSCT on transplantation outcomes

**Figure 2:**
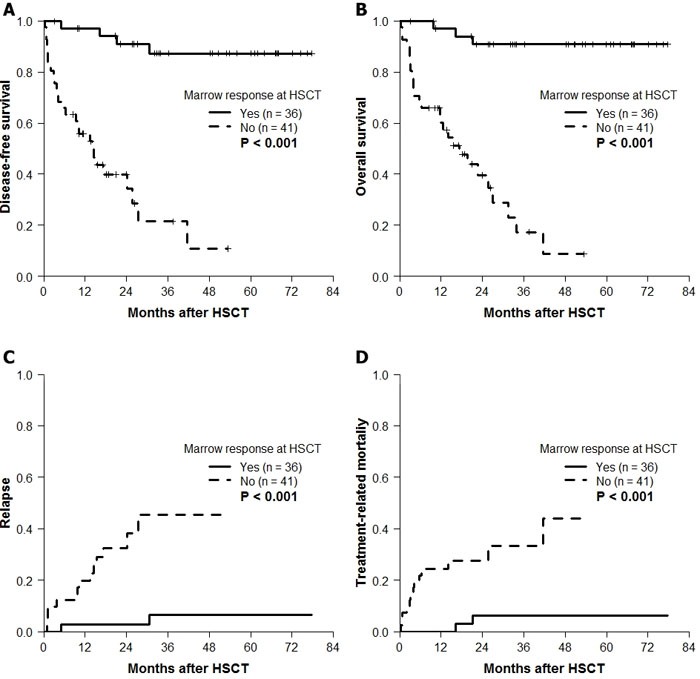
Kaplan-Meier analyses for DFS according to HMT response (*n* = 77) DFS according to **A**. the best response and **B**. the final response to HMT.

**Figure 3:**
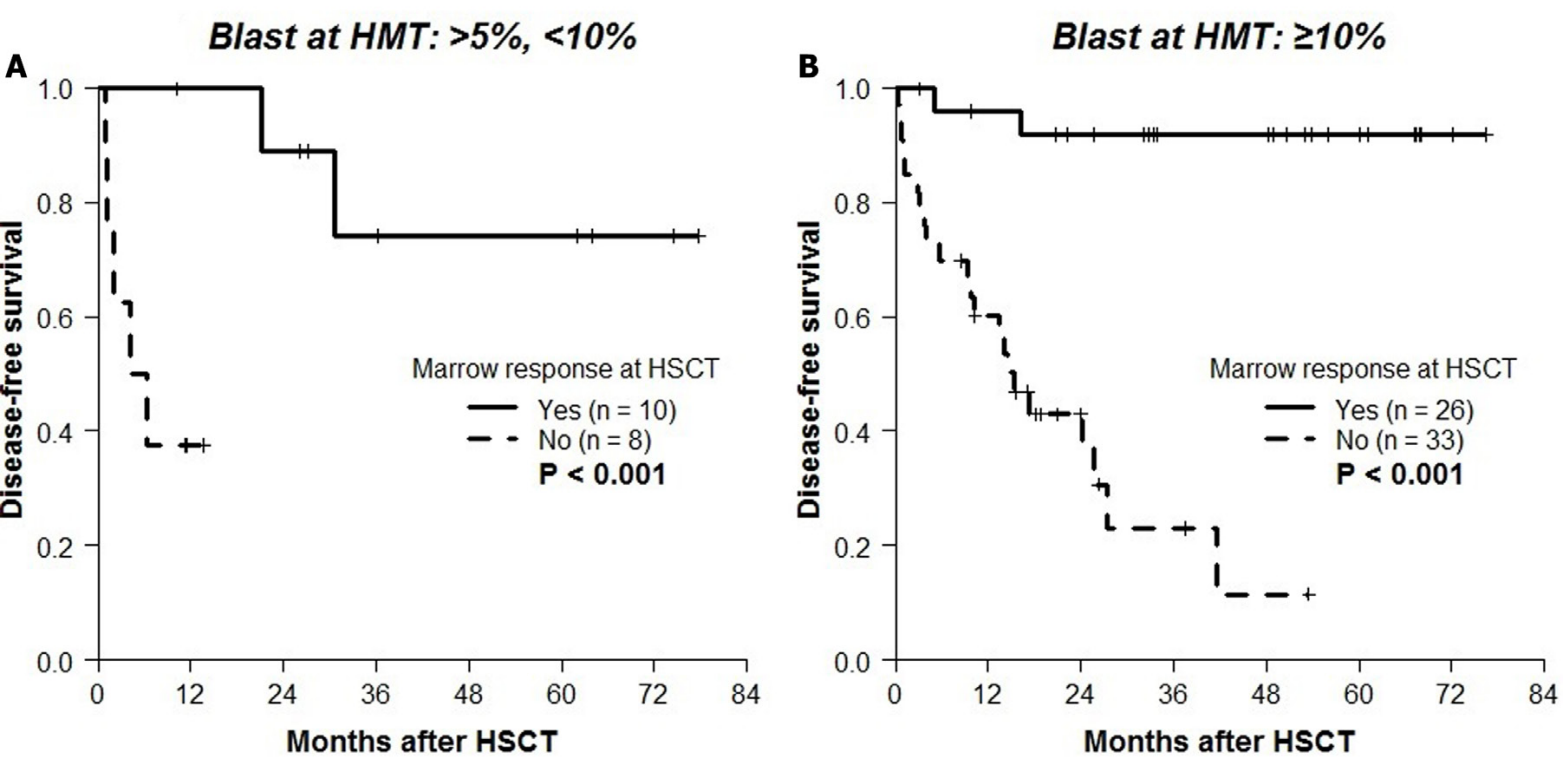
Posttransplantation outcome according to continued marrow response to HMT at HSCT (*n* = 77) We dichotomized patients into two groups according to the achievement of marrow response that sustained until the time of transplantation: marrow response (*n* = 36) and no marrow response (*n* = 41). The two groups demonstrated significant differences in the probabilities of **A**. DFS (87.3% ± 6.0% *vs*. 10.7% ± 8.8%), **B**. OS (90.9% ± 5.0% *vs*. 8.6% ± 7.4%), **C**. cumulative incidence of relapse (6.5% ± 4.6% *vs*. 45.4% ± 10.9%), and **D**. cumulative incidence of TRM (6.2% ± 4.3% *vs*. 43.9% ± 13.7%).

**Figure 4:**
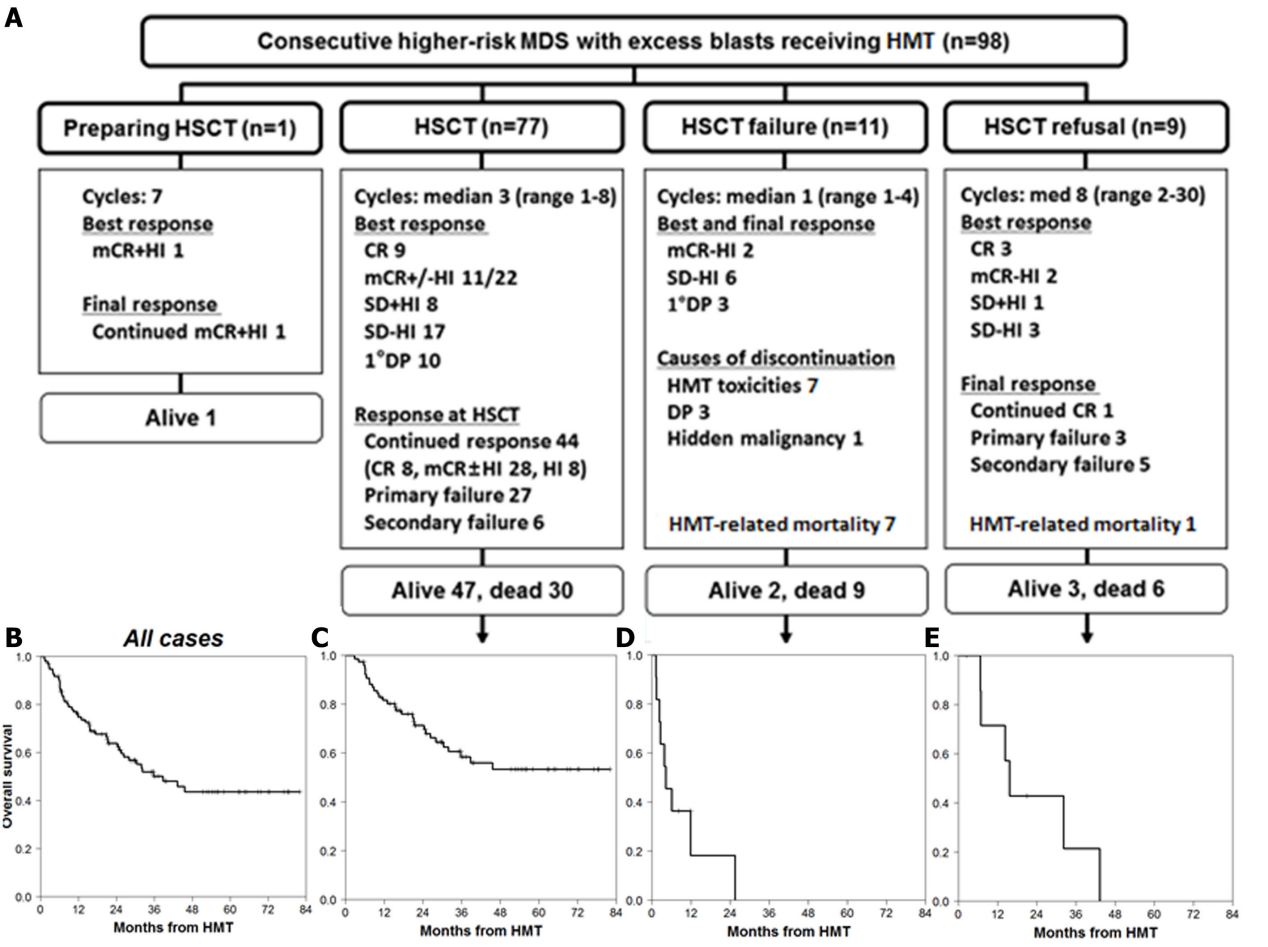
Kaplan-Meier analyses for DFS according to marrow response to HMT at HSCT in patient groups subdivided by marrow blast at HMT DFS according to marrow response at HSCT in **A**. 18 patients with blast counts of >5% and < 10% (marrow response: 88.9% ± 10.5% *vs*. no marrow response: 37.5% ± 17.1%, *P* = 0.003) and in **B**. 59 patients with blast counts of ≥10% blasts (91.7% ± 5.6% *vs*. 9.5% ± 8.1%, *P* < 0.001).

The 4-year post-transplant DFS according to the best response (Figure 2A) or the final response at HSCT (Figure 2B) after HMT was different. Based on the poor median DFS of patients with secondary failure (9.3 months), the final response at HSCT was chosen to further evaluate the effect of HMT response on transplantation outcomes. Observing significantly better DFS rates in patients with CR (100%), mCR+HI (80.8%), and mCR-HI (85.9%) at HSCT, the response at HSCT was dichotomized into “marrow response” (*n* = 36) and “no marrow response” (*n* = 41). The baseline characteristics of these two groups are compared in Table 1. The two groups demonstrated significant differences in the 4-year probabilities of DFS (*P* < 0.001; Figure 3A), OS (*P* < 0.001; Figure 3B), CIR (*P <* 0.001; Figure 3C), and CITRM (*P* = 0.001; Figure 3D). When similar analyses were performed according to marrow blast count at HMT, significantly better DFS rates were observed among patients with blast levels of >5% and < 10% (marrow response: 88.9% ± 10.5% *vs*. no marrow response: 37.5% ± 17.1%, *P* = 0.003; Figure 4A) and patients with blast levels of ≥10% (91.7% ± 5.6% *vs*. 9.5% ± 8.1%, *P* < 0.001; Figure 4B). Table 2 lists the influence of variables on transplant outcomes in univariate analysis. In multivariate analysis (Table 3), no marrow response at HSCT was a significant predictor for inferior OS (hazard ratio [HR]: 12.6, *P* < 0.001), DFS (HR: 10.2, *P* < 0.001), CIR (HR: 8.5, *P =* 0.004), and CITRM (HR: 8.6, *P* = 0.002). In addition, poor cytogenetic risk [15] at HSCT was a significant predictor of inferior OS (HR: 2.9, *P* = 0.018), DFS (HR: 3.7, *P* = 0.003), and CIR (HR: 5.0, *P* = 0.002). Based on these results, we evaluated the combined value of cytogenetic risk and continued marrow response for predicting HSCT outcomes, illustrated in Supplementary Figure 1A-1D.

**Table 1:** Clinical characteristics of transplanted patients

Variable	All (*n* = 77)	Marrow response (*n* = 36)	No marrow response (*n* = 41)	P
Age at HMT (years); median (range)	51 (21–65)	46 (21–65)	54 (25–64)	0.005
Age at HSCT (years); median (range)	51 (21–65)	46 (21–65)	55 (26–65)	0.009
Sex, *n* (%)				
Male,	48 (62.3)	16 (44.4)	32 (78.0)	
Female	29 (37.7)	20 (55.6)	9 (22.0)	0.002
Days from higher-risk diagnosis to HMT, median (range)	19.0 (1–180)	12 (1–180)	21 (1–106)	0.356
Days from HMT to HSCT, median (range)	128 (46–377)	121 (46–335)	135 (63–377)	0.564
Hypomethylating agents				
Azacitidine	50 (64.9)	20 (55.6)	30 (73.2)	
Decitabine	27 (35.1)	16 (44.4)	11 (26.8)	0.106
Cycles of HMT before HSCT, median (range)	3 (1–8)	3 (1–8)	3 (1–6)	0.466
WHO classification at HMT, *n* (%)				
RAEB-1	16 (20.8)	10 (27.8)	6 (14.6)	
RAEB-2	59 (76.6)	25 (69.4)	34 (82.9)	
CMMoL-1	1 (1.3)	-	1 (2.4)	
CMMoL-2	1 (1.3)	1 (2.8)	-	0.358
Hemogram at HMT, median (range)				
WBC, x 10^9^/L	2.41(0.26–74.4)	2.54 (0.78–36.5)	2.32 (0.26–74.4)	0.593
ANC, x 10^9^/L	0.83(0.03–16.8)	0.89 (0.07–13.5)	0.79 (0.03–16.8)	0.272
Hemoglobin, d/gL	8.4(3.5–15.5)	8.4 (3.8–11.9)	8.4 (3.5–15.5)	0.451
Platelet, x 10^9^/L	62 (5–883)	68 (5–339)	59 (5–883)	0.226
BM blast at HMT, median (range)	12.0 (5.8–19.1)	12.0 (6.0–19.1)	13.0 (5.8–19.0)	0.613
IPSS cytogenetic risk at HMT, *n* (%)				
Good	35 (45.5)	15 (41.7)	20 (48.8)	
Intermediate	25 (32.5)	13 (36.1)	12 (29.3)	
Poor	17 (22.1)	8 (22.2)	9 (22.0)	0.686
Transfusion dependency at HSCT, *n* (%)				
Yes	45 (58.4)	16 (44.4)	29 (70.7)	
No	32 (41.6)	20 (55.6)	12 (29.3)	0.020
Donor/recipient sex combination, *n* (%)				
Female ♢ Male	16 (20.8)	5 (13.9)	11 (26.8)	
Others	61 (79.2)	31 (86.1)	30 (73.2)	0.163
Donor type, *n* (%)				
HLA-matched sibling donor	35 (45.5)	15 (41.7)	20 (48.8)	
HLA-matched unrelated donor	17 (22.1)	9 (25.0)	8 (19.5)	
Partially HLA-mismatched unrelated donor	6 (7.8)	4 (11.1)	2 (4.9)	
Haploidentical related donor	19 (24.7)	8 (22.2)	11 (26.8)	0.885
Conditioning intensity, *n* (%)				
MAC	54 (70.1)	24 (66.7)	30 (73.2)	
RIC	23 (29.9)	12 (33.3)	11 (26.8)	0.534
HMT response at HSCT, *n* (%)				
CR	8 (10.4)	8 (22.2)	-	
mCR+HI	11 (14.3)	11 (30.6)	-	
mCR-HI	17 (2.1)	17 (47.2)	-	
SD+HI	8 (10.4)	-	8 (19.5)	
SD-HI	17 (22.1)	-	17 (41.5)	
Primary DP	10 (13.0)	-	10 (24.4)	
Secondary failure	6 (7.8)	-	6 (14.6)	NE

HSCT, hematopoietic stem cell transplantation; HMT, hypomethylating treatment; WHO, World Health Organization; RAEB-1, refractory anemia of excess blast -1; RAEB-2, refractory anemia of excess blast-2; CMMoL-1, chronic myelomonocytic leukemia-1; CMMoL-2, chronic myelomonocytic leukemia-2; WBC, white blood cell; ANC, absolute neutrophil count; BM, bone marrow; IPSS, International Prognostic Scoring System; MAC, myeloablative conditioning; RIC, reduced-intensity conditioning; CR, complete remission; mCR, marrow complete remission; SD, stable disease; HI, hematologic improvement; DP, disease progression.

* The WHO diagnoses in all patients (*n* = 98), including those not receiving HSCT (*n* = 21), were RAEB-1 (*n* = 20), RAEB-2 (*n* = 73), CMMoL-1 (*n* = 1), and CMMoL-2 (*n* = 4).

**Table 2:** Univariate analysis of factors affecting the 4-year OS, DFS, CIR and CITRM after transplantation

		OS		DFS		CIR		CITRM	
Variables	No.	%	P	%	P	%	P	%	P
Response at HSCT			<0.001		<0.001		<0.001		<0.001
Marrow response	36	90.9 ± 5.0		87.3 ± 6.0		6.5 ± 4.6		6.2 ± 4.3	
No marrow response	41	8.7 ± 7.4		10.7 ± 8.8		45.4 ± 10.9		43.9 ± 13.7	
Drugs			0.077		0.145		0.447		0.342
Decitabine	27	66.2 ± 10.2		62.0 ± 10.5		25.9 ± 7.3		18.0 ± 9.0	
Azacitidine	50	45.8 ± 8.6		48.5 ± 8.3		25.9 ± 7.3		25.7 ± 7.1	
Age at HSCT, years			0.049		0.047		0.184		0.278
<52	39	63.7 ± 8.7		65.1 ± 8.4		16.7 ± 6.4		18.1 ± 7.1	
≥52	38	43.5 ± 9.6		40.3 ± 9.7		33.5 ± 9.6		26.3 ± 8.0	
Sex			0.013		0.025		0.219		0.128
Female	29	75.2 ± 9.2		71.6 ± 9.5		18.1 ± 8.7		10.3 ± 8.3	
Male	48	40.5 ± 8.6		42.4 ± 8.5		27.1 ± 6.9		30.5 ± 5.8	
IPSS at start of HMT			0.283		0.343		0.630		0.532
Intermediate-2	56	59.0 ± 7.3		57.9 ± 7.2		22.7 ± 6.3		19.4 ± 6.3	
High	21	39.6 ± 13.6		42.1 ± 13.4		27.4 ± 11.3		30.5 ± 13.4	
IPSS cytogenetic risk at HSCT			0.004		0.001		0.002		0.873
Good/Intermediate	64	62.4 ± 7.1		60.8 ± 7.2		14.5 ± 5.4		22.0 ± 6.1	
Poor	13	23.1 ± 11.7		23.1 ± 11.7		53.9 ± 15.0		23.1 ± 12.7	
Transfusion dependency at HSCT			0.006		0.002		0.002		0.517
Yes	45	41.5 ± 8.1		40.2 ± 8.0		35.5 ± 7.6		24.3 ± 7.1	
No	32	73.4 ± 9.1		75.0 ± 9.1		5.0 ± 5.0		20.0 ± 8.4	
Donor/recipient sex combination			0.976		0.983		0.545		0.535
Female ♢ Male	16	50.3 ± 15.6		53.4 ± 7.2		28.0 ± 12.7		13.7 ± 9.4	
Others	61	54.5 ± 7.2		58.3 ± 13.3		22.6 ± 6.0		24.1 ± 6.2	
Donor type*			0.757		0.684		0.180		0.441
Conventional	52	41.7 ± 30.4		49.7 ± 8.3		28.8 ± 7.1		21.5 ± 7.0	
Alternative	25	51.3 ± 8.3		60.6 ± 10.5		13.9 ± 7.8		25.5 ± 9.4	
Conditioning Intensity			0.561		0.518		0.256		0.769
RIC	23	60.3 ± 11.2		61.9 ± 10.8		15.1 ± 8.4		23.0 ± 9.4	
MAC	54	51.0 ± 8.2		49.4 ± 8.2		27.9 ± 7.0		22.7 ± 7.0	
HCT-CI risk			0.156		0.109		0.539		0.071
Low/ Intermediate	45	59.3 ± 8.5		61.7 ± 8.1		21.3 ± 7.0		15.0 ± 5.9	
High	32	43.2 ± 10.3		38.7 ± 10.6		27.2 ± 8.6		36.6 ± 10.6	

OS, overall survival; DFS, disease-free survival; CIR, cumulative incidence of relapse; CITRM, cumulative incidence of treatment-related mortality; HSCT, hematopoietic stem cell transplantation; IPSS, International Prognostic Scoring System; HMT, hypomethylating treatment; RIC, reduced-intensity conditioning; MAC, myeloablative conditioning; HCT-CI, hematopoietic cell transplant- co-morbidity index.

*Conventional donor included HLA-matched sibling or unrelated donor; Alternative donor included partially HLA-mismatched or haploidentical related donor.

**Table 3:** Multivariate analysis of factors affecting the 4-year OS, DFS, CIR and CITRM after transplantation

	OS		DFS		CIR		CITRM	
Variables	HR (95% CI)	P	HR (95% CI)	P	HR (95% CI)	P	HR (95% CI)	P
Response at HSCT								
Marrow response	1		1		1		1	
No marrow response	12.6 (3.5–45.4)	<0.001	10.2(3.3–31.8)	<0.001	8.5 (2.0–36.7)	0.004	8.6 (2.2–34.2)	0.002
IPSS cytogenetic risk at HSCT								
Good/Intermediate	1		1		1		-	
Poor	2.9 (1.2–6.9)	0.018	3.7 (1.5–8.7)	0.003	5.0 (1.8–13.8)	0.002		
Age at HSCT, years								
<52	1		1		-		-	
≥52	2.3 (1.0–5.4)	0.066	2.3 (1.0–5.4)	0.055				
HCT-CI risk								
Low/ Intermediate	-		-		-		1	
High							2.5 (0.9–6.9)	0.068
Sex								
Male	1		1		-		-	
Female	0.9 (0.3–2.2)	0.745	0.9 (0.4–2.2)	0.876				
Transfusion dependency at HSCT								
Yes	1		1		-		-	
No	2.2 (0.8–6.0)	0.109	2.6 (1.0–6.7)	0.055				

OS, overall survival; DFS, disease-free survival; CIR, cumulative incidence of relapse; CITRM, cumulative incidence of treatment-related mortality; HR, hazard ratio; CI, confidence interval; HSCT, hematopoietic stem cell transplantation; IPSS, International Prognostic Scoring System; HCT-CI, hematopoietic cell transplant- co-morbidity index.

### Predictors of marrow response or TRM after HMT

Univariate and multivariate analyses of the impact of clinical variables at HMT on achievement of marrow response and HMT-related mortality in entire cohort (*n* = 98) are summarized in Supplementary Table 1. The presence of peripheral blood (PB) blasts was associated with unfavorable marrow response compared to patients without PB blasts (odds ratio: 0.33, 95% CI: 0.12-0.93, *P* = 0.04). None of the variables was independently associated with HMT-related mortality, although poor cytogenetic risk was a potential prognostic factor. These unfavorable impacts of PB blasts were further translated into differences in 4-year OS in entire cohort (20.5% ± 11.0% *vs*. 52.0% ± 6.9%, *P =* 0.002; Supplementary Figure 2A) as well as in transplant recipients (22.0% ± 12.6% *vs*. 61.7% ± 7.1%, *P* < 0.001; Supplementary Figure 2B).

## DISCUSSION

Advanced disease status at HSCT has a clear negative effect on the post-transplantation relapse rate, which is particularly evident among patients with higher-risk MDS [16, 17]. Although AML-type induction chemotherapy to reduce disease burden before transplant could be used aiming at reducing relapse rates [18], the role of pre-HSCT intensive therapy remains controversial [19, 20], as the improved relapse rates may be offset by significant toxicities. Furthermore, the related analyses supporting pre-HSCT induction chemotherapy are subject to an inherent selection bias, being related to the considerable proportion of patients with induction chemotherapy rendered ineligible for HSCT [21]. Therefore, the selection of pre-transplant bridging regimen should be essentially focused on preventing disease progression and toxicity that would interfere in proceeding to HSCT, while inducing disease “debulking” (ideally within a few months) translating into improved transplant survival.

Although HMT is reported to have low toxicity, there are no reports regarding its toxicity during preparation for transplantation and the subsequent HSCT success rate. Therefore, to provide clinically relevant information, and to minimize selection bias, we screened all patients who received HMT for higher-risk MDS with excess blasts, and only excluded patients who were ineligible for HSCT. Our analyses revealed that bridging failure occurred in 11% of the patients with pre-HSCT HMT, and that 8% experienced HMT-related mortality. These results compare favorably with the higher mortality rate of 16% during the induction chemotherapy among carefully screened patients who were to undergo HSCT in a prospective clinical trial [22]. The relatively low HMT-related mortality rate in our study suggested that HMT is a feasible pre-transplant option.

Disease stage at the time of transplantation has been known as one of the most important factors that influence outcome after allogeneic HSCT [17, 18, 23]. However, a recent study by Potter et al. using a large registry data suggested that, unlike for patients with primary refractory disease, the post-transplant outcomes of patients with MDS not in CR were not significantly worse than those in CR [24]. This is in line with our results which showed that continued marrow response even without HI at HSCT was an independent predictor of a favorable prognosis, and that CR was not a prerequisite for prolonged survival. Furthermore, the acquisition of HI only without marrow response did not translate into a survival benefit in our transplantation setting. This finding is interesting, because it conflicts with the survival benefit from the achievement of HI in the HMT-only setting [25]. Similarly, in our previous report, the positive effect of marrow response with or without HI after HMT on post-transplant DFS was mainly evident in patients classified as higher-risk MDS before HMT while achievement of HI was the main response type in lower-risk MDS [11], suggesting that the influences of HMT response at HSCT vary according to pre-HMT risk groups. The controversies regarding the relationship between HMT response and post-transplantation survival may be explained by the heterogeneity in the time of response assessment, whether or not HI is included for definition of overall response, and different response patterns according to pre-HMT risk groups [9, 11, 26]. Future studies are needed to determine the role of pre-transplant achievement of HI by HMT in comparison with upfront HSCT, as worsening of cytopenias from pre-HSCT HMT aimed to achieve HI in patients with low blast counts may increase their risk of infectious complications [27].

The present study revealed that 50 of the 98 cases experienced marrow response after a median of 2 cycles (range: 1-9 cycles), and that the cumulative marrow response rates after 2 cycles, 3 cycles, and 4 cycles were 68%, 84%, and 96%, respectively. The relatively high marrow response rate in our study may be associated with the frequent marrow assessments. Moreover, the frequent response evaluation was helpful for immediately identifying patients who had achieved a short-term marrow response followed by a rapid increments in marrow blasts. In fact, secondary failure typically occurred at approximately 1 month after achieving marrow response, and 5 of the 6 patients with secondary failure died due to relapse (*n* = 3) or TRM (*n* = 2). These findings suggest that it is important to maintain continued marrow response until HSCT. According to the interim results of a recent prospective Italian multicenter trial which was designed to complete at least 4 cycles of pre-HSCT azacitidine treatment before transplant, approximately 48% of the enrolled patients could not proceed to HSCT due to reasons including disease relapse/ progression (35%) or adverse events (27%) [28]. Thus, unlike in non-transplantation setting [25, 29], once marrow response has been achieved, immediate HSCT seems most effective to increase the likelihood of cases with higher-risk MDS and excess blasts reaching transplantation with favorable post-transplant outcomes, rather than to continue on HMT beyond time of marrow response. Moreover, response to HMT may reflect the innate sensitivity of the MDS clone, and HMT-induced drug resistance or selection of a resistant clone should be considered, as it may become more likely after continued treatment [30]. The influence of mCR upon survival was recently suggested in a study of decitabine treatment [31], although our results may provide the first evidence regarding the importance of marrow response in enhancing HSCT outcomes for higher-risk MDS with excess blasts.

Our strategy of pre-transplant “debulking” treatment with hypomethylating agent that was immediately followed by HSCT seems to provide favorable post-transplant outcomes. The median survival duration was 39.3 months after HMT among all patients in this study, which is superior to the reported 18-25 months after azacitidine or 5-day decitabine treatment [21, 32–34], showing the well-known effect of HSCT in higher-risk MDS [35]. And also, the beneficial role of HSCT might have been enhanced by pre-HSCT HMT, as our 4-year DFS rate after HSCT (54%) was superior to those from previous studies (15-30%) [4]. Furthermore, although we only evaluated patients with higher-risk MDS and excess blasts (>5%), the survival rate was comparable to that observed in recent HSCT analyses encompassing both lower- and higher-risk MDS [8, 10, 36], suggesting that HMT pre-treatment before HSCT is feasible. We also assume that, for those patients with lack of a matched related or unrelated donor, our approach of immediately proceeding to haploidentical-related transplant may have contributed to the favorable outcomes in our study [3, 37].

Previous studies have reported that HMT response could be predicted by several clinical parameters, such as white blood cell count, platelet count, karyotype, and marrow blast count [13, 38, 39]. In agreement with a recent study conducted on a large registry data [40], the presence of PB blasts was the only independent predictor of poor marrow response, which was associated with inferior survival in our study. Thus, while pre-HSCT HMT could be preferred in patients without PB blasts, up-front HSCT or pre-HSCT intensive chemotherapy could be considered for patients with PB blasts. In addition to marrow response at HSCT, poor karyotype was also a risk factor for relapse and poor survival. Thus, marrow response, PB blast detection, and karyotyping may be useful for selecting pre-HSCT treatment option and patients who are most appropriate for HSCT. Mutations in certain genes may be useful for prediction of posttransplantation survival, particularly in patients with complex karyotypes (e.g., by assessing *TP53*) [41, 42], although these markers require further studies for prediction of HMT response [39, 43–45]. Future clinical trial might be useful for developing and evaluating a stepwise decision-making model that is based on these risk factors.

In conclusion, the findings of our study indicate that the sequential treatment using HMT followed immediately by HSCT is feasible and offers an efficient treatment strategy for higher-risk MDS patients with excess blasts. Notably, the achievement of marrow response by pre-HSCT HMT and proceeding to transplant while maintaining the response was significantly associated with lower incidences of relapse and TRM and improved DFS. These results suggest that, once marrow response is achieved during HMT, immediate HSCT rather than continuing HMT should be considered for patients with higher-risk MDS and excess blasts. The development of clinical and molecular tools for earlier recognition of HMT response may also optimize selecting transplant candidates and timing. Whether pre-HSCT HMT offers advantages over upfront HSCT and intensive chemotherapy in higher-risk patient, especially those with excess blasts, needs to be confirmed by randomized trials.

## PATIENTS AND METHODS

### Patient selection

All adult patients with MDS who were eligible for HSCT were retrospectively screened to identify cases with higher-risk MDS and excess marrow blasts that received pre-HSCT HMT between January 2009 and June 2015 at the Catholic Blood and Marrow Transplantation Center (Seoul, Korea). We analyzed the patients receiving HMT for MDS with marrow blast levels of >5% and intermediate-2 or high-risk group according to the International Prognostic Scoring System [15], regardless of the patient's intention to undergo HSCT. The selection criteria for HSCT eligibility included an age of ≤65 years, an ECOG performance status of ≤2, and no major organ failure. This study was approved by the institutional review board of the Seoul St. Mary's Hospital at the Catholic University of Korea, and complied with the tenets of the Declaration of Helsinki.

### Treatment strategy and procedures

All patients received a standard HMT regimen of azacitidine (75 mg/m^2^/day for 7 days) or decitabine (20 mg/m^2^/day for 5 days). According to our institution's guidelines regarding HSCT for higher-risk MDS, transplantation was performed immediately after a donor was available, regardless of the treatment cycle or response to HMT. When patients experienced secondary AML after HMT, induction chemotherapy was administered when patients were judged to be able to tolerate the intensive treatment. For conventional donor HSCT, the patients underwent a preparative regimen of fludarabine (150 mg/m^2^) with 2 days or 4 days of intravenous busulfan (3.2 mg/kg/day) and rabbit anti-thymocyte globulin (ATG; 5-10 mg/kg; Genzyme, Cambridge, MA). For haploidentical related donor transplantation, the patients underwent regimens of fludarabine (150 mg/m^2^) with 2 days of intravenous busulfan (3.2 mg/kg/day), total body irradiation (800 cGy or 400 cGy), and ATG (5.0 mg/kg). GVHD prophylaxis was performed using short-course methotrexate (10 mg/m^2^ intravenous bolus on days +1, +3, +6, and +11) plus cyclosporine for related donor HSCT or tacrolimus for unrelated/haploidentical donor HSCT. The protocol for the transplantation procedures was the same, with the exception of the conditioning step and general transplantation procedures being performed as previously described [11, 46]. All of the transplanted patients received PB stem cells.

### Definitions

Bone marrow aspiration and biopsy were performed after every 2 HMT cycles, based on the clinical needs, and immediately before conditioning. Hematological responses were only assessed when bone marrow was obtained, and HI was assessed whenever complete blood cell counting was performed, using the International Working Group 2006 response criteria [47]. The best and final responses (at HSCT) were defined as CR, partial remission, mCR+HI, mCR-HI or SD+HI. Patients with CR/mCR with or without HI were designated as marrow response group. Non-responders and patients who had failed to maintain a previous response at the time of the HSCT were categorized as primary failure (SD-HI or primary DP) and secondary failure (loss of response or relapse), respectively [48]. Myeloid and platelet engraftment was defined as the first of 3 consecutive days with an absolute neutrophil count (ANC) of ≥ .5 × 10^9^/L and the first of 7 consecutive days with a platelet count ≥ 20 × 10^9^/L without transfusion, respectively. GVHD was diagnosed and graded according to the clinical consensus criteria [49, 50]. The hematopoietic cell transplantation-comorbidity index was estimated according to Sorror et al. [51]. HMT-related mortality was arbitrarily defined as death that occurred due to any event during the 42 days after the final administration of HMT, in the absence of DP, which was assessed using marrow blast counts.

### Statistical analyses

Differences in the categorical and continuous variables among the patient risk subgroups were compared using the χ2 test, Fisher's exact test, or Mann-Whitney's test, as appropriate. Time to event was assessed from the infusion day. Events for DFS were relapse or death from any cause, whereas death from any cause was a relevant event for OS. TRM was defined as death from any cause during continuous remission after HSCT. Survival curves for OS and DFS were plotted using the Kaplan-Meier method, and differences were evaluated using the log-rank test. The cumulative incidence of TRM and relapse were plotted and compared using the Gray test. The effects of the covariates on OS and DFS were determined using the Cox proportional hazard model. Factors were considered significant if they exhibited a *P*-value of < 0.05 in a two-tailed likelihood ratio test. The effects of the covariates on the CIR and CITRM were determined using the semi-parametric proportional hazard model for the sub-distribution of competing risks. The final models were created using the backward conditional method. Most statistical analyses were performed using SPSS software (version 13.0; SPSS Inc., Chicago, IL, USA), and the cumulative incidence analyses were performed using R software (http://cran.r-project.org/).

## SUPPLEMENTARY MATERIALS FIGURES AND TABLES


