# Supplementary material for: Better transplant outcome with pre-transplant marrow response after hypomethylating treatment in higher-risk MDS with excess blasts
Source: Oncotarget. 2016 Oct 6;8(7):12342–54. doi: 10.18632/oncotarget.12511 (PMC5355349; doi:10.18632/oncotarget.12511)
Supplement: Supplementary file 1 [file oncotarget-08-12342-s001.pdf]

Better transplant outcome with pre-transplant marrow response after hypomethylating treatment in higher-risk MDS with excess blasts

Supplementary Material

Supplementary Table 1
Prognostic factors of marrow response achievement and HMT-related mortality

	Achievement of marrow response					HMT-related mortality				
	Univariate analysis			Multivariate analysis		Univariate analysis			Multivariate analysis	
	E/N	%	P	OR (95% CI)	P	E/N	%	P	OR (95% CI)	P
Age										
<53	29/49	59.2				2/49	4.1			
>53	21/49	42.9	0.16			6/49	12.2	0.27		
Sex										
Male	26/61	42.6		1		4/37	10.8			
Female	24/37	64.9	0.04	0.53 (0.22–1.29)	0.17	4/61	6.6	0.47		
Drug										
Decitabine	19/32	59.4				3/32	9.4			
Azacitidine	31/66	47.0	0.18			5/66	7.6	0.71		
MDS type										
De novo	48/90	53.3				7/90	7.8			
Secondary	2/8	25.0	0.12			1/8	12.5	0.51		
WHO diagnosis										
RAEB-1	14/20	70.0				3/20	15.0			
RAEB-2	33/73	45.2				5/73	6.8			
CMMoL-1/2	3/5	60.0	0.12			0/5	0	0.59		
IPSS										
Intermidiate-2	37/70	52.9				5/70	7.1			
High	13/28	46.4	0.36			3/28	10.7	0.69		
Cytogenetic risk										
Good/Intermediate	41/75	54.7				3/75	4.0		1	
Poor	9/23	39.1	0.24			5/23	21.7	0.02	4.12 (0.78–21.78)	0.10
Normal karyotype										
Yes	18/38	44.7				2/38	5.3			
No	33/60	55.0	0.41			6/60	10.0	0.49		
WBC (x10^6/L)										
< 3000	30/54	55.6				2/54	3.7			
≥ 3000/	20/44	45.5	0.42			6/44	13.6	0.14		
ANC (x10^6/L)										
< 1000	30/52	57.7				2/52	3.8			
≥ 1000	20/46	43.5	0.22			6/46	13.0	0.14		
Hemoglobin (g/dL)										
< 10	44/80	55.0				7/80	8.8			
≥ 10	6/18	33.3	0.12			1/18	5.6	1.0		
Platelets (x10^9/L)										
< 100	35/73	47.9				7/73	9.6			
≥ 100	15/25	60.0	0.36			1/25	4.0	0.46		
PB blast										
No	43/73	58.9		1		4/74	5.4		1	
Yes	7/25	28.0	0.01	0.33 (0.12–0.93)	0.04	4/24	16.7	0.1	3.36 (0.64–17.80)	0.15

BM blast						
< 15%	32/57	56.1		4/57	7.0	
≥ 15%	18/41	439	0.31	4/41	9.8	0.72

HMT, hypomethylating treatment; OR, odds ratio; CI, confidence interval; MDS, myelodysplastic syndrome; WHO, World Health Organization; RAEB-1, refractory anemia of excess blast -1; RAEB-2, refractory anemia of excess blast -2; CMMoL -1/2, chronic myelomonocytic leukemia -1 & -2; IPSS, International Prognostic Scoring System; WBC, white blood cell; ANC, absolute neutrophil count; PB, peripheral blast; BM, bone marrow

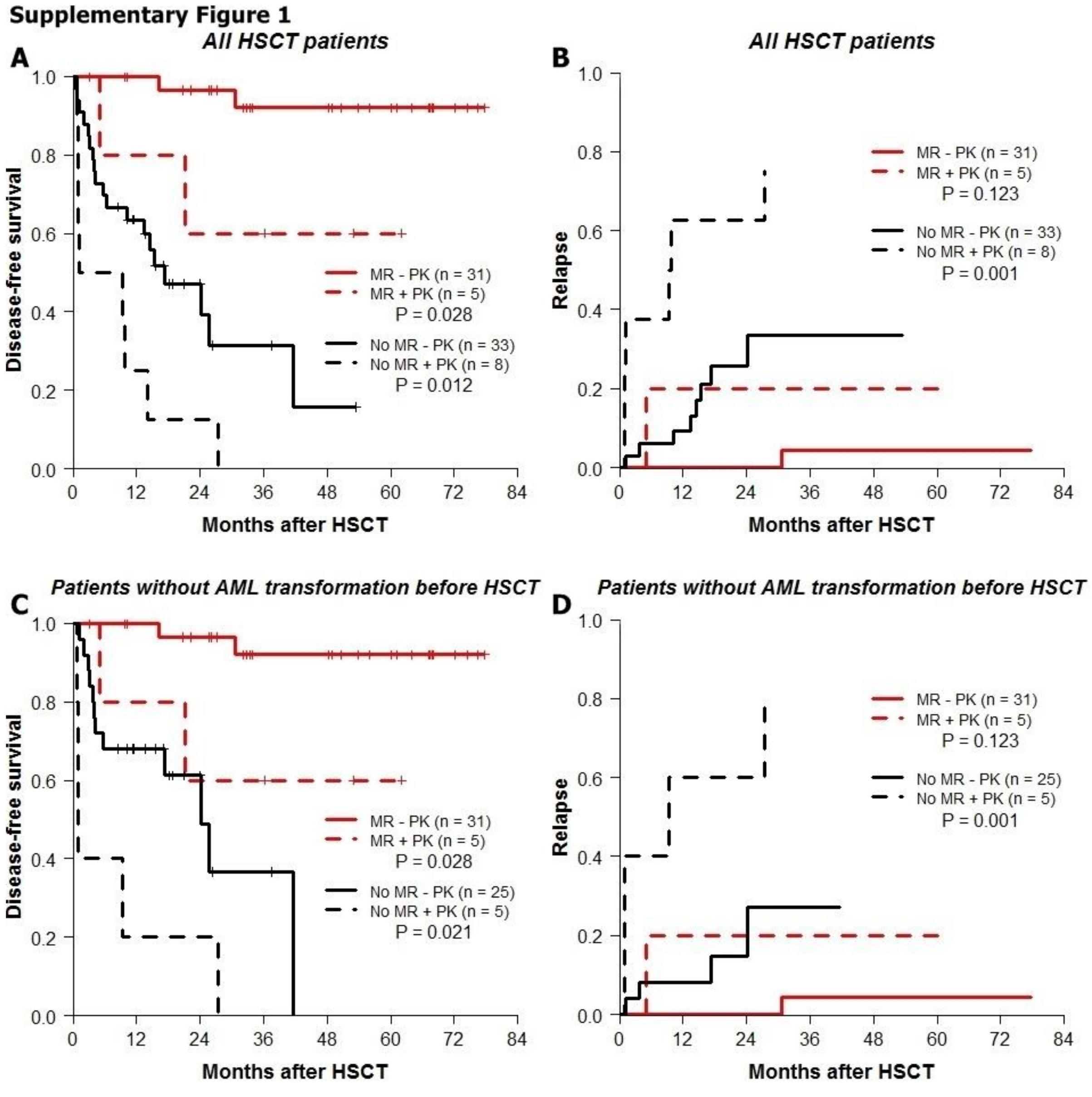

Supplementary Figure 1. Posttransplantation outcome according to combined risk groups of marrow response (MR) and poor karyotype (PK). Probability of (A) DFS and (B) cumulative incidence of relapse in all HSCT patients (n = 77); probability of (C) DFS and (D) cumulative incidence of relapse in 66 HSCT patients who did not experience AML transformation before HSCT.

Supplementary Figure 2

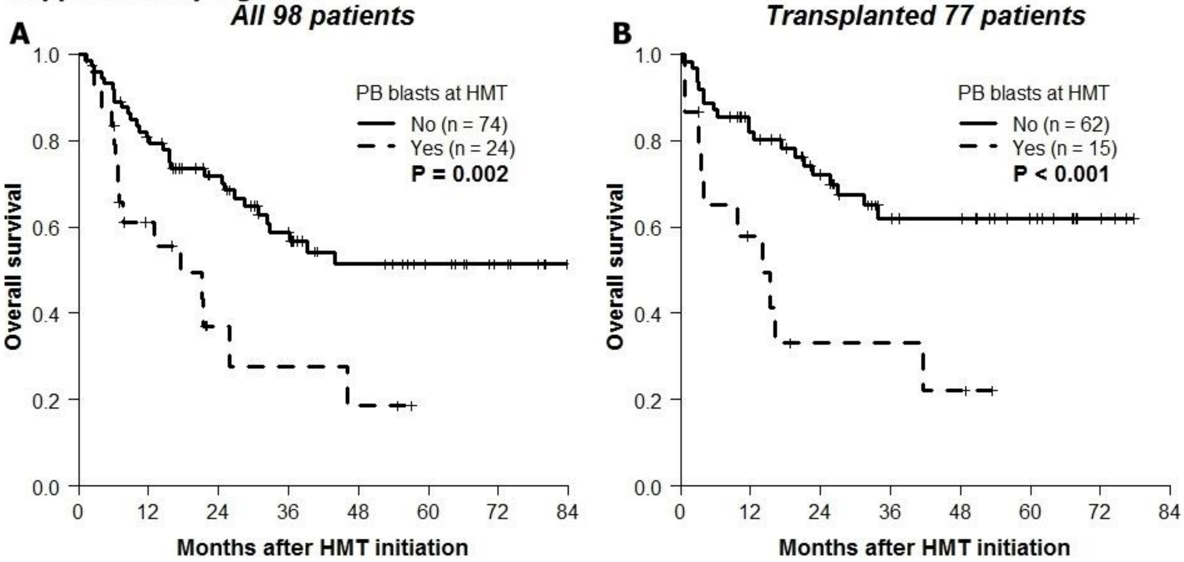

Supplementary Figure 2. Kaplan-Meier analyses for survival from HMT according to the presence of PB blasts at HMT. The influences of PB blasts on HMT response and leukemic transformation were further translated into significant differences in 4-year OS from HMT according to the presence of PB blasts among (A) all higher-risk MDS patients who received HMT (n = 98; 20.5% ± 11.0% vs. 52.0% ± 6.9%) and among (B) those who received HSCT following HMT (n = 77; 22.0% ± 12.6% vs. 61.7% ± 7.1%).
